# Biostimulant Potential of Aquatic Plants: Investigating *Egeria densa* and Other Macrophytes’ Potential in Crop Growth

**DOI:** 10.3390/plants14071018

**Published:** 2025-03-25

**Authors:** Diego Munhoz Gomes, Raphael Mereb Negrisoli, Alysson Dias Dalmas, Renato Nunes Costa, Mariana Bueno Domingues, Ramon Hernany Gomes, Maria Lúcia Bueno Trindade, Eduardo Heraldo, Caio Antonio Carbonari, Edivaldo Domingues Velini

**Affiliations:** 1 Plant Protection Department, São Paulo State University, Botucatu 18610-034, SP, Brazil; d.gomes@unesp.br (D.M.G.); alysson.dalmas@unesp.br (A.D.D.); renato.costa@unesp.br (R.N.C.); caio.carbonari@unesp.br (C.A.C.); edivaldo.velini@unesp.br (E.D.V.); 2 AquaPlant Pesquisa, Desenvolvimento e Inovação, Botucatu 18605-525, SP, Brazil; 3 Bioativa-Pesquisas Estratégicas em Biociência, Botucatu 18605-525, SP, Brazil; mbdomingues9@gmail.com (M.B.D.); r.gomes@unesp.br (R.H.G.); 4 Auren Energia, São Paulo 05425-070, SP, Brazil; eduardo.heraldo@aurenenergia.com.br

**Keywords:** plant growth promotion, agriculture, sustainability, alternative fertilization, extraction solutions

## Abstract

This study investigates the potential of macrophytes as biostimulants in agricultural applications through a two-stage experimental approach. In the first stage, a screening experiment evaluated 12 macrophyte species using ethanolic and potassium chloride extracts at two doses (1 and 5 kg fresh biomass/ha) applied to bioindicator species *Cucumis sativus* (C3) and *Urochloa decumbens* (C4). Controlled greenhouse conditions and randomized block designs ensured reliability. Dry biomass was measured 21 days after treatment (DAT), revealing varied macrophyte effects. Ethanolic extracts of *Typha domingensis* and *Egeria densa* demonstrated significant biomass increases, particularly for *U. decumbens*, while potassium chloride extracts often reduced biomass. *E. densa* was selected for further analysis due to its promising results and ease of selective harvesting. In the second stage, a dose–response experiment assessed the impact of *E. densa* ethanolic extracts on *Phaseolus vulgaris* at six doses (0.25 to 4 kg fresh biomass/ha). Optimal results were observed at 1–2 kg/ha, yielding 15% increases in plant height and dry biomass. Higher doses showed diminishing returns. These findings highlight the potential of *E. densa* as a sustainable biostimulant and a solution for macrophyte overabundance in Brazilian reservoirs, supporting agricultural and environmental objectives.

## 1. Introduction

Aquatic plants in the watersheds of Brazilian rivers and reservoirs have become an increasing environmental challenge, particularly with invasive species [1]. In recent years, the proliferation of these macrophytes has been attributed to an excess of nutrients, such as nitrogen and phosphorus, which drive uncontrolled growth and promote eutrophication in the reservoir or rivers [2]. This overgrowth obstructs water flow, hampers navigation, and negatively impacts hydroelectric plants [3,4]. Moreover, the decomposition of aquatic plants reduces dissolved oxygen levels, threatening the survival of aquatic organisms and diminishing local biodiversity [5].

The phenomenon of eutrophication exacerbates these impacts, as elevated nutrient levels promote aquatic vegetation growth, including microalgae capable of producing toxic substances such as microcystin. This toxin compromises water quality, posing risks to both ecosystems and human health. To preserve aquatic ecosystems and ensure a supply of potable water, efficient nutrient management and invasive plant control are essential [6,7,8,9].

Nevertheless, there are challenges involved in controlling aquatic plants, particularly with the prohibition of pesticides in Brazilian rivers and reservoirs. The primary technique adopted is mechanical removal, which, while effective, has limitations such as high costs and difficulties in keeping pace with plant growth [10]. This imbalance calls for alternative solutions to optimize management and reduce environmental impacts.

In this scenario, while macrophytes are often viewed as a problem, they can represent an opportunity if their characteristics are harnessed. Mechanical removal of these plants, for instance, not only contributes to restoring aquatic ecosystems but can also yield renewable resources. In addition, these macrophytes could be used to produce biomass, biofuels, fertilizers, and other sustainable products, transforming an environmental challenge into a valuable resource [11].

In the example of algae and seaweed extracts [12], the use of aquatic plants for biostimulant production emerges as a promising and sustainable alternative. The biostimulant market has been growing, leveraging substances and microorganisms to enhance the performance of agricultural and forestry crops. Macrophytes, in particular, may contain promising bioactive compounds that can be utilized to develop biostimulants. Integrating these plants into the agricultural market not only mitigates environmental impacts but also contributes to proper species management [13,14,15].

Biostimulants can be defined as materials, other than fertilizers, that can promote plant growth in low doses [16]. They play an essential role in sustainable agriculture by boosting productivity without relying on chemical inputs. These compounds influence various physiological functions of plants, such as germination, rooting, growth, and resistance to abiotic stresses. Commonly used components include humic acids, seaweed extracts, and microorganisms, which improve soil structure, nutrient availability, and plant health [17,18].

To use macrophytes as biostimulants, an effective method for extracting beneficial compounds is essential to enhance crop growth, yield, or fruit quality. Therefore, the extraction technique of bioactive compounds plays a crucial role in ensuring the final product’s efficacy. Various extraction methods exist, including organic solvents, aqueous solutions, and supercritical fluid extraction. Solvent extraction, using substances like ethanol and acetone, is effective for obtaining both lipophilic and hydrophilic compounds [16,17]. Aqueous extraction, which uses water as a solvent, is simple and ideal for hydrosoluble compounds. Supercritical fluid extraction, employing carbon dioxide (CO_2_), is highly selective and efficient, enabling the extraction of diverse compounds without leaving residues [19,20,21].

After producing the macrophyte extracts, the compound solution can be applied in various ways, including foliar applications, soil applications, or irrigation. Foliar application ensures rapid absorption by plants, while soil application provides a more gradual effect, fostering root growth. Irrigation with biostimulants, on the other hand, allows for continuous and efficient compound distribution [22].

The hypothesis is that many macrophytes found worldwide have the potential to serve as effective biostimulants, as in the example found in algae, offering a sustainable solution for their utilization while addressing the challenge of high infestation. Additionally, repurposing these macrophytes could create economic opportunities for communities living near rivers and reservoirs. Furthermore, as ethanolic extracts are used commonly in the literature, it is expected that these would produce the best results.

Thus, this study’s objective is to investigate macrophytes with potential for biostimulant production, evaluating their bioactive properties and optimal extraction techniques. Utilizing these plants in biostimulant development offers an innovative and ecological solution, contributing both to environmental preservation and sustainability in agriculture. 

## 2. Results

### 2.1. Screening Study

There was no effect of experimental run across all experiments; therefore, all data were pooled and analyzed. Furthermore, there were no interactions between extraction solution and dose; thus, the results are presented separately for each extraction solution. The impact of macrophyte ethanolic extract doses on *C. sativus* and *U. decumbens* is summarized in Table 1 and Table 2, respectively.

In the case of *C. sativus*, a significant effect was observed with the *Typha domingensis* ethanolic extract, where a 12% increase in dry biomass was recorded at a dose of 5 kg of fresh biomass ha^−1^. In contrast, no significant differences were detected between treated and untreated plants for all other macrophyte extracts and doses.

Macrophyte ethanolic extracts presented more pronounced effects on *U. decumbens*. For *E. densa*, a dose of 1 kg ha^−1^ resulted in a 10% increase in dry biomass, while a dose of 5 kg ha^−1^ led to a smaller increase of 4.5%, compared to the untreated control. In contrast, extracts from *Ludwigia peploides* exhibited a dose-dependent response, with a 10% inhibition in dry biomass observed at 1 kg ha^−1^ but a 22% increase at 5 kg ha^−1^. Similarly, *Eichhornia crassipes* extracts stimulated plant growth at both doses, producing approximately a 10% increase in dry biomass. Lastly, the application of *Alternanthera sessilis* extract at 1 kg ha^−1^ resulted in a 12% increase in biomass, whereas a higher dose of 5 kg ha^−1^ caused a 5% reduction in biomass.

The effects of macrophyte potassium chloride (KCl) extract doses on *C. sativus* and *U. decumbens* are summarized in Table 3 and Table 4, respectively. Similar to the ethanolic extracts, the KCl extracts had more pronounced effects on *U. decumbens* compared to *C. sativus*. For *C. sativus*, only *L. peploides* exhibited a significant effect, leading to a 16% increase in dry biomass at a dose of 5 kg ha^−1^. In contrast, the KCl extracts significantly impacted *U. decumbens*, particularly with extracts from *E. densa*, *L. peploides*, *Polygonum hydropiperoides*, *E. crassipes*, and *T. domingensis*. Overall, these extracts tended to reduce biomass rather than stimulate growth. For instance, *E. densa* caused a 12% decrease in biomass at 1 kg ha^−1^. Similarly, *L. peploides* and *P. hydropiperoides* resulted in biomass reductions of 8% and 10% at doses of 1 kg ha^−1^ and 5 kg ha^−1^, respectively. Among the extracts, only *E. crassipes* and *T. domingensis* showed a stimulatory effect, each increasing biomass by 8% at 1 kg ha^−1^. However, this positive response was reversed at higher doses, with both species exhibiting approximately a 10% biomass reduction at 5 kg ha^−1^.

Considering *E. densa* as one of the most promising candidates, the physicochemical parameters of *Egeria densa* extracts were measured to compare ethanolic and KCl extracts (Table 5). Ethanolic extract has a higher pH (7.05) compared to the KCl extract (6.24). In general, ethanol extracted more amino acids and hormones than KCl extracts. Furthermore, asparagine is the most abundant amino acid in both extracts but is significantly higher in the ethanolic extract (416.05 ppm) than in the KCl extract (8.04 ppm). Moreover, ethanolic extracts resulted in greater aspartic acid in comparison to KCl extracts, with 3000% more aspartic acid than KCl extracts. Other amino acids such as leucine, valine, and phenylalanine are more evenly distributed between the extracts. Both extracts have relatively low total lipid content (0.0091 g/100 mL for ethanol, 0.0072 g/100 mL for KCl), and total sugars are slightly lower in the KCl extract (0.039%) than in the ethanolic extract (0.042%).

**Figure 1:**
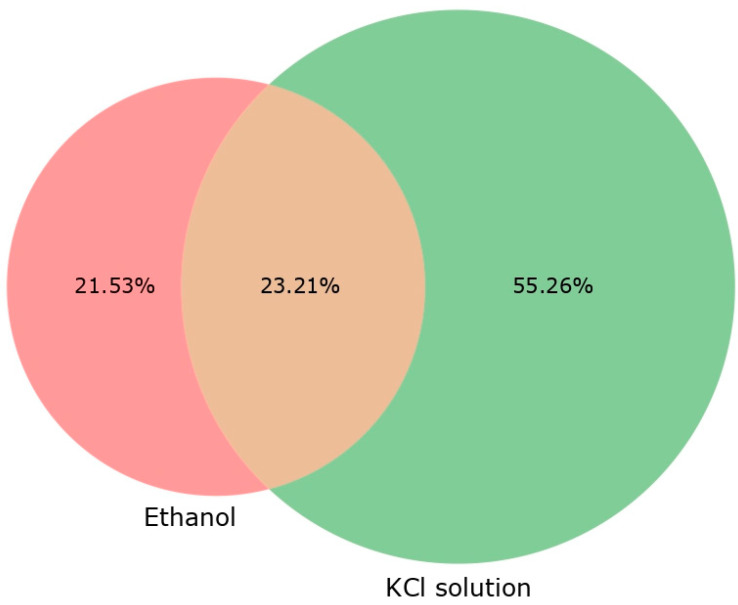
Venn diagram with percentage of compounds analyzed in QTOF for ethanolic and KCl extracts. Percentage values: compounds for each extract subtracted from blank compounds for the graphical analysis.

In addition to measuring the physicochemical parameters of Egeria densa extracts in KCl and ethanol, an analysis in QTOF was conducted to assess the potential of each extract in positive and negative ionization (Table 6). The KCl extraction resulted in more detected substances, higher signal intensities, and larger chromatographic areas, indicating it may be more efficient at extracting a broader range of compounds (Figure 1). The KCl solution extracted over 35% more compounds than the ethanolic extracts. However, the ethanolic extracts exhibited higher retention times (RTs) with lower m/z ratios. Despite these differences, the total chromatographic area was similar for both extraction solutions. The Venn diagram showed that 52% of the compounds were extracted using KCl, compared to 21.53% with the ethanolic extraction.

### 2.2. Dose–Response Curve with Egeria densa Ethanolic Extracts in Common Beans

The preliminary results using *E. densa* extracts demonstrated promising outcomes. Specifically, ethanolic extracts showed a notable increase in efficacy at a concentration of 1 kg ha^−1^, despite a reduction in effectiveness at 5 kg ha^−1^. These findings highlight the need for a more comprehensive investigation, including a dose–response analysis of *E. densa* ethanolic extract, to fully explore its potential as a plant stimulant and to establish the optimal application dose.

**Figure 2:**
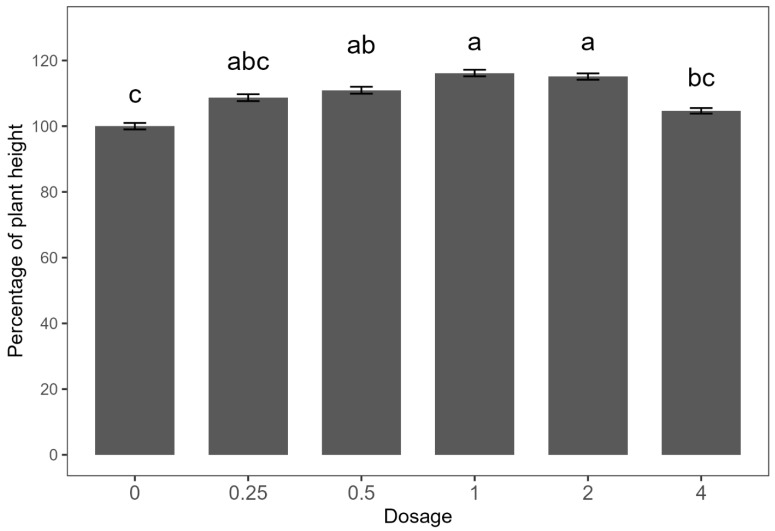
Dose–response curve of *Egeria densa* ethanolic extracts against common-bean height. Means within bars followed by the same letter are not significantly different according to an LSD test at the 5% level of significance. Error bars indicate ± standard error (SE).

Since no significant differences were observed between the two experimental runs, the data were combined for analysis. The dose–response effect on plant height did not fit the Mitscherlich model; thus, the data are presented in a bar plot (Figure 2). Consistent with findings from the preliminary study, treatments with *E. densa* fresh biomass at 1 and 2 kg ha^−1^ resulted in the greatest increases in plant height, approximately 15% higher than the untreated control. The 0.5 kg ha^−1^ treatment yielded a moderate increase of 10%, while no significant differences were observed with the 0.25 and 4 kg ha^−1^ treatments compared to the control.

**Figure 3:**
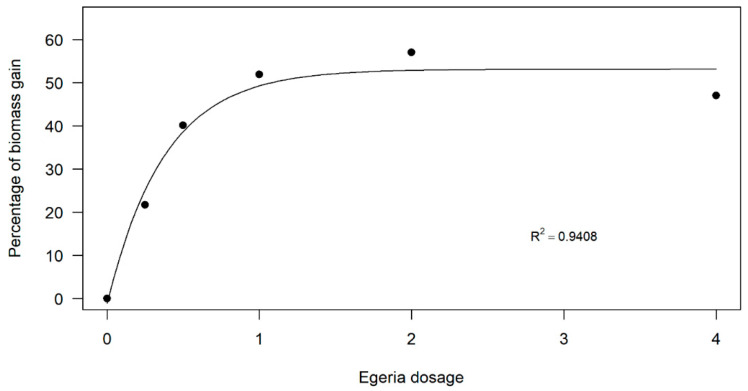
Dose–response curve of *Egeria densa* ethanolic extracts in common-bean dry biomass production.

A similar pattern was evident for dry biomass production (Figure 3). The dose–response curve indicated increased biomass production at intermediate doses. However, while the highest dose (4 kg ha^−1^) still resulted in greater biomass production than the control, the curve showed a decline, suggesting the presence of an upper dose threshold, beyond which biomass production diminishes.

## 3. Discussion

The aim of this study is to find a solution for the accumulation of macrophytes within Brazilian reservoirs, enabling the use of these macrophytes as plant stimulants in agricultural crops. Thereafter, the macrophytes used in this investigation are troublesome macrophytes, i.e., with high-density populations spread worldwide [23,24]. Nevertheless, the majority of species from this study are found at the water surface (floating or emerging macrophytes), and a few are submerged macrophytes: *E. densa* and *Hydrilla verticillata* [25].

The responses of the two bioindicator species, *C. sativus* and *U. decumbens*, varied depending on the macrophyte species. While there were no significant differences between the extract solutions, the responses of both bioindicators to the macrophytes’ effects differed. Overall, *U. decumbens* proved to be a more sensitive bioindicator for the macrophytes tested, exhibiting amplified responses compared to *C. sativus*. Notably, potassium chloride extracts from several macrophytes tended to reduce biomass production, in contrast to ethanolic extracts, which on average resulted in growth effects. The species-specific responses highlight how different macrophytes may promote or suppress biomass production in target plants. Some macrophytes (*Ludwigia peploides* in *C. sativus*, *Eichhornia crassipes* in *U. decumbens*) have dose-dependent effects, which could be relevant for biostimulant or allelopathic research.

Among the macrophytes evaluated in this study, *E. densa* stands out as one of the most found species in Brazilian reservoirs. Its unique characteristics make it particularly suitable for harvesting: as a fully submerged plant, it allows for selective harvesting of a single species rather than a mixture of macrophytes. In contrast, surface-dwelling macrophytes are typically found as part of a mixed community, posing challenges for species-specific collection.

The preliminary results using *E. densa* extracts demonstrated promising outcomes. Specifically, ethanolic extracts showed a notable increase in efficacy at a concentration of 1 kg ha^−1^, despite a reduction in effectiveness at 5 kg ha^−1^. These findings highlight the need for a more comprehensive investigation, including a dose–response analysis of *E. densa* ethanolic extract, to fully explore its potential as a plant stimulant and to establish the optimal application dose.

Therefore, considering its facilitation in collection in addition to its high density in Brazilian reservoirs, *E. densa* was selected for the following in-depth study to determine its potential as a biostimulant. Nevertheless, *L. peploides* emerged as another macrophyte with promising results, indicating its potential use as a crop biostimulant. However, its low occurrence during collection and frequent mixing with other species led this study to prioritize the investigation of *E. densa* over *L. peploides*.

Considering *E. densa*’s physicochemical parameters, ethanolic extracts indicate a more neutral nature, whereas the KCl extract is slightly more acidic. In general, ethanolic extracts resulted in more interesting extractions than KCl extraction. In addition, the higher m/z and lower RT in KCl extracts suggest that it favors the extraction of more polar and potentially heavier compounds compared to ethanol. However, ethanol extraction may preferentially extract less polar compounds, which could account for the higher average RT, including hydrophobic amino acids or lipophilic substances. A more detailed study should be conducted in the future for metabolomic analysis to identify which compounds are the most responsible for plant biomass improvement. However, as ethanolic extraction resulted in greater biomass production, this indicates that greater compound extraction is not necessarily a good indicator, as amino acids and hormones have proven to be a better indication. Nevertheless, ethanolic extract contains higher amino acid concentrations, which might be useful for bioactive compound studies and may corroborate with the results found in this screening study.

QTOF analysis allowed us to analyze the amount of compounds that were extracted from the *E. densa* extraction solutions. Ethanol extraction may preferentially extract less polar compounds, which could account for the higher average RT, including hydrophobic amino acids or lipophilic substances. A more detailed study should be conducted in the future for metabolomic analysis to identify which compounds are most responsible for plant biomass improvement.

The dose–response curve indicated increased biomass production at intermediate doses. However, while the highest dose (4 kg ha^−1^) still resulted in greater biomass production than the control, the curve showed a decline, suggesting the presence of an upper dose threshold beyond which biomass production diminishes.

Research on utilizing macrophytes is very limited, with few studies exploring their potential as green manure [26] or biofertilizers [27]. However, no studies have specifically examined the macrophytes tested in this study in the context of extraction using ethanol or KCl, highlighting a gap and an opportunity for further research. In contrast, algae extracts have long been established in the agricultural market, consistently demonstrating significant improvements in crop yield and even crop protection [18].

When *Kappaphycus alvarezii* was applied to the leafy vegetable Amaranthus polygamous, using a 10% leaf extract combined with 50% conventional fertilization, researchers observed a 20% increase in dry biomass production compared to the treatment with 100% conventional fertilization [28]. Two types of seaweed were also evaluated as biostimulants in maize cultivation [29]. Laminaria and *Ascophyllum nodosum* spp. enhanced crop growth, particularly by improving root development and nutrient uptake, suggesting their effectiveness in promoting resilience to abiotic stress.

Leveraging macrophytes like *Egeria densa* presents an excellent opportunity for growers, not only because it may enhance crop production but also because it may serve as a potential solution for managing invasive populations of this species in reservoirs. Removing *E. densa* from water bodies could benefit the ecosystem by reducing nutrient levels and mitigating factors contributing to eutrophication. Additionally, harvesting portions of the plant would allow it to regrow over time, maintaining its utility, highlighting the significant role of *E. densa* in removing nitrogen from water, which is critical for ecosystem health [30].

Among aquatic plants with potential for biostimulants are seaweeds such as *Ascophyllum nodosum* and *Ecklonia maxima*. The former, rich in plant hormones, minerals, and amino acids, is highly valued in the market [31,32]. Meanwhile, *E. maxima*, known for its resilience to adverse environmental conditions, contains compounds like polyphenols and fucoidans, which have antioxidant properties and other benefits for plant growth [33,34,35]. Similarly to algae, further studies are required for the understanding of *E. densa* and other aquatic plants with beneficial properties that provide crop stimulation.

Furthermore, *E. densa* is relatively easier to harvest compared to other macrophytes, as it typically remains submerged in dense clusters and is often found as a single-species stand, streamlining collection efforts. Future research should focus on evaluating the effects of *E. densa* extracts on various crops, with particular emphasis on comparing their impact on C3 and C4 species. Additionally, further studies are needed to elucidate the mechanisms through which *E. densa* positively influences crops such as common beans and *U. decumbens*, their interpretation, and the experimental conclusions that can be drawn.

## 4. Materials and Methods

The experiments were conducted in two distinct stages. The first experiment aimed to perform an initial screening to select macrophytes with potential biostimulant properties and determine the most efficient extraction phase for obtaining plant extracts. The second experiment involved creating a dose–response curve using the most promising macrophyte selected from the first experiment. All experiments were repeated twice (experimental runs) and conducted in a controlled greenhouse environment (25 °C ± 2).

### 4.1. Initial Screening

The experiments were designed using a randomized block design, using 12 macrophytes species in two extract solutions and two doses, with five replicates. The experimental units consisted of 1.7 L pots filled with Carolina Soil^®^ substrate (Carolina Soil, Pardinho, Brazil), composed of sphagnum peat moss, vermiculite, and carbonized rice husks, with a pH of 5.7 (±0.5). The initial screening utilized two crop species, *Cucumis sativus* and *Urochloa decumbens*, as bioindicators to assess the effects of macrophytes. These species were chosen for their sensitivity to exogenous applications and because they represent different photosynthetic pathways, i.e., those of C3 and C4 plants, respectively.

Macrophytes were collected from two distinct reservoirs (Nova Avanhandava and Bariri Reservoirs, Tietê River in São Paulo, Brazil) and cleaned with running water; this was followed by root removal. All 12 species used in this experiment comprehend troublesome aquatic plants that present high population density and are problematic for water quality or energy production (Table 7).

**Figure 4:**
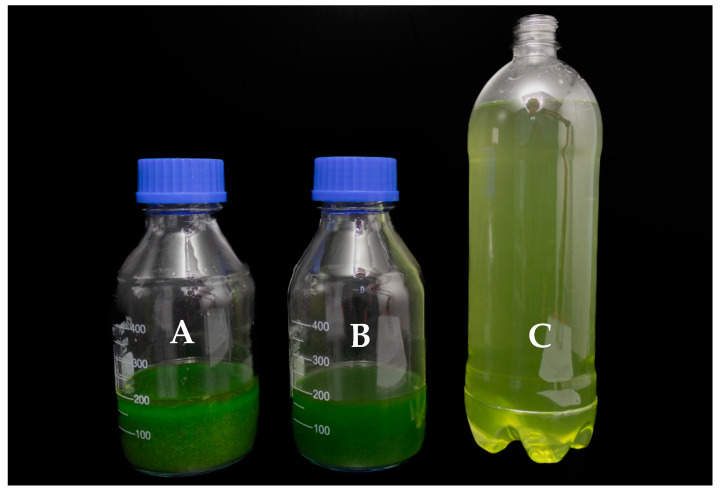
Macrophyte extract processing and preparation: extract after processing with the extractant solution (**A**); extract after filtration (**B**); application mixture ready for treatment (**C**).

The two extraction solutions were 92.8% ethanol and 16% KCl as extractants, that is, macrophyte extracts produced with ethanolic and potassium chloride extracts. For this screening study, two doses were analyzed to determine preliminary impacts, using 1 and 5 kg of fresh biomass per hectare. For extract preparation, the collected samples were homogenized; this was followed by the separation of 100 g of fresh material. The plants were ground with 200 mL of the extractant solution and then filtered to remove solid particles. The final extract volume was adjusted to 300 mL by adding more extractant solution. This procedure was performed for both extractant solutions (Figure 4).

Applications on *C. sativus* were carried out at the V3 phenological stage, while for *U. decumbens*, these were carried out when the plants reached three tillers. The applications were conducted using a stationary automated sprayer installed in a controlled environment. The system was equipped with speed, pressure, and flow control features. The sprayer was fitted with four XR 11002 nozzles. The speed was maintained at 1 m s^−1^ with a pressure of 2 bar, resulting in an application volume of 200 L ha^−1^.

To assess the effects of macrophytes on the bioindicators, dry biomass was evaluated 21 days after treatment (DAT). This process was performed after harvesting the plants, drying them in a temperature-controlled oven at 60 °C for 15 days, and weighing them using an analytical balance with a precision of 0.1 milligrams. Based on the results from the screening study, an in-depth analysis was conducted using the most promising macrophyte to determine the best dose and its effectiveness on a third crop. For that, a dose–response curve was used in the second experiment.

Extracts of *E. densa* in ethanol and KCl were analyzed using LC-MS coupled with QTOF (Shimadzu LCMS-9030, Kyoto, Japan) for compound identification and polarization assessment. This analysis aimed to compare the differences between ethanolic and KCl extractions, focusing on the quantity and types of compounds extracted.

### 4.2. Dose–Response Curve with Egeria densa

After the screening analysis, the treatment with *E. densa* was chosen for further in-depth experiments. Thus, this experiment was conducted using a dose–response curve with *E. densa* extract to determine the ideal dose and its effect on common beans (*Phaseolus vulgaris* L.) (Table 8). Experimental units and treatment application were performed as described in Section 2.1. Nevertheless, treatments were carried out at the V3 phenological stage.

Height evaluations were conducted at 21 DAT. Measurements were taken from the base of the stem at the substrate level to the tip of the longest mature leaf. At the end of the experiment, at 21 DAT, the plants were harvested, dried in a temperature-controlled oven (60 °C) for 15 days, and weighed on an analytical balance with a precision of 0.1 milligrams.

### 4.3. Data Analysis

For the screening study, dry biomass data for macrophytes in two doses and two extracts solutions were subjected to ANOVA to test the interaction between macrophytes doses and extract solutions. Treatment means were separated using an LSD test at the 0.05 level of confidence using a package. The results for each bioindicator and each macrophyte were analyzed separately. If the difference between the experimental runs were not significant, the data were analyzed combined. All analyses were performed using R statistical software [36] with the packages agricolae and ggplot.

Dose–response analysis was performed with the package drc [37] by correlating *E. densa* dose with common-bean height and dry biomass production. When the dose–response curve did not fit, the data were subjected to ANOVA and means separated using an LSD test at the 0.05 level of confidence. Data for common-bean height were transformed into percentage of the untreated control, and dry biomass was transformed into biomass gain in comparison to the untreated control, with the untreated control set at 0% gain over 21 DAT. The data were fit to non-linear Mitscherlich regression models, using Y = a [1−10(−c(X + b))] [38]. The parameters a, b, and c correspond to the equation’s coefficients, where parameter a is the maximum asymptote of the curve and represents the maximum quantities of dry biomass gain (%). The lateral shift of the curve corresponds to parameter b and its concavity to parameter c. The value of Y indicates the total dry biomass gain (%), and X represents the *E. densa* doses (kg of fresh biomass ha^−1^).

## 5. Conclusions

This study highlights *E. densa* ethanolic extract as a promising candidate for agricultural biostimulants, demonstrating growth-promoting effects at optimal doses, especially on *P. vulgaris* and *U. decumbens*. This approach also addresses the ecological challenges posed by invasive macrophyte populations. This study’s focus on species such as *Egeria densa*, which is problematic in many regions, aligns with global efforts to manage invasive species while promoting sustainable agricultural practices. Ethanolic extracts of *E. densa* resulted in greater amino acid extraction, which may lead to improved plant biomass production. Ethanolic *E. densa* extract dose–response showed the extract’s great potential to be used as crop stimulant, especially in doses with 2 kg of fresh biomass ha^−1^.

Future studies are recommended to uncover the biochemical mechanisms underlying the stimulatory effects of *E. densa* extracts on plants and assess the long-term environmental impacts and scalability of harvesting *E. densa* for agricultural applications. Additionally, *L. peploides* has shown potential as a promising candidate for further investigation among the other macrophytes tested.

## 6. Patents

This research is part of the P&D ANEEL project by Auren Energia and Bioativa—Pesquisas Estratégicas em Biociências, “Exploração Sustentável de Compostos Naturais”.

## Figures and Tables

**Table 1:** Effect of ethanolic macrophyte extracts in two doses (1 and 5 kg fresh biomass ha^−1^) in *Cucumber sativus* dry biomass production.

Treatment	Dose ^a^		F-Test
	1 kg	5 kg	
	% of Untreated		
*Egeria densa*	94.0	103.8	0.543 ns
*Ludwigia peploides*	108.5	85.8	2.884 ns
*Polygonum hydropiperoides*	94.3	92.2	0.528 ns
*Polygonum lapathifolium*	105.8	97.1	0.549 ns
*Eichhornia crassipes*	92.7	105.2	0.963 ns
*Hydrilla verticillata*	95.8	95.0	0.355 ns
*Alternanthera sessilis*	90.3	95.5	0.387 ns
*Commelina diffusa*	96.5	102.1	0.206 ns
*Brachiaria subquadripara*	96.2	97.4	0.111 ns
*Paspalum repens*	95.5	95.3	0.217 ns
*Enydra anagallis*	98.6	90.6	0.769 ns
*Typha domingensis*	93.4	112.1	2.816 *

^a^—kg of macrophyte fresh biomass per hectare; *—significant difference with LSD test at 5% probability; ns—non-significant with LSD test at 5% probability.

**Table 2:** Effect of ethanolic macrophyte extracts in two doses (1 and 5 kg fresh biomass ha^−1^) in the *Urochloa decumbens* dry biomass production.

Treatment	Dose ^a^		F-Test
	1 kg	5 kg	
*Egeria densa*	110.7	104.5	2.498 *
*Ludwigia peploides*	90.5	122.1	3.158 *
*Polygonum hydropiperoides*	107.8	110.4	1.457 ns
*Polygonum lapathifolium*	101.3	100.8	0.119 ns
*Eichhornia crassipes*	108.5	111.1	3.081 *
*Hydrilla verticillata*	109.4	106.4	1.996 ns
*Alternanthera sessilis*	111.7	95.8	3.153 *
*Commelina diffusa*	105.2	111	0.838 ns
*Brachiaria subquadripara*	106.7	106.3	1.539 ns
*Paspalum repens*	98	111.9	0.681 ns
*Enydra anagallis*	106.6	105.5	0.563 ns
*Typha domingensis*	98.6	97.7	0.036 ns

^a^—kg of macrophyte fresh biomass per hectare; *—significant difference with LSD test at 5% probability; ns—non-significant with LSD test at 5% probability.

**Table 3:** Effect of potassium chloride macrophyte extracts in two doses (1 and 5 kg fresh biomass ha^−1^) in the *Cucumber sativus* dry biomass production.

Treatment	Dose ^a^		F-Test
	1 kg	5 kg	
	% of Untreated		
*Egeria densa*	102.2	96.4	0.188 ns
*Ludwigia peploides*	96.6	116.0	2.209 *
*Polygonum hydropiperoides*	110.4	93.5	1.001 ns
*Polygonum lapathifolium*	108.3	103.4	0.220 ns
*Eichhornia crassipes*	102.5	97.5	0.136 ns
*Hydrilla verticillata*	98.4	99.1	0.045 ns
*Alternanthera sessilis*	92.6	101.1	0.553 ns
*Commelina diffusa*	100.8	98.3	0.061 ns
*Brachiaria subquadripara*	105.8	101.5	0.112 ns
*Paspalum repens*	95.3	93.2	0.338 ns
*Enydra anagallis*	108.4	111.6	0.494 ns
*Typha domingensis*	86.2	103.2	1.476 ns

^a^—kg of macrophyte fresh biomass per hectare; *—significant difference with LSD test at 5% probability; ns—non-significant with LSD test at 5% probability.

**Table 4:** Effect of potassium chloride macrophyte extracts in two doses (1 and 5 kg fresh biomass ha^−1^) in the *Urochloa decumbens* dry biomass production.

Treatment	Dose ^a^		F-Test
	1 kg	5 kg	
*Egeria densa*	88.8	98.1	1.925 *
*Ludwigia peploides*	92.4	89.7	2.414 *
*Polygonum hydropiperoides*	86.2	88.9	2.139 *
*Polygonum lapathifolium*	99.5	92.5	1.663 ns
*Eichhornia crassipes*	107.2	86.9	2.363 *
*Hydrilla verticillata*	96.4	93.9	0.469 ns
*Alternanthera sessilis*	90.9	110.2	1.844 ns
*Commelina diffusa*	105.8	95.3	1.114 ns
*Brachiaria subquadripara*	91.4	90.3	0.848 ns
*Paspalum repens*	96.3	105.1	0.674 ns
*Enydra anagallis*	94.5	103.9	0.246 ns
*Typha domingensis*	108.3	90.4	4.54 *

^a^—kg of macrophyte fresh biomass per hectare; *—significant difference with LSD test at 5% probability; ns—non-significant with LSD test at 5% probability.

**Table 5:** Physicochemical parameters of *Egeria densa* extracts in KCl and ethanol.

Physicochemical Parameters	Ethanolic Extract	Potassium Chloride Extract
Electrical Conductivity (µS/cm)	483.95	152.5
pH	7.05	6.24
Amino Acids and Hormones	ppm	
Alanine	14.64	0.38749
Arginine	0.8647	Traces
Asparagine	416.0518	8.04312
Aspartic Acid/Aspartate	28.8956	0.76951
Cystine	Traces	Traces
Glutamic Acid	13.0079	0.28459
Glutamine	0.8755	0.36196
Glycine	1.06235	Traces
Histidine	3.5874	Traces
Isoleucine	6.5893	5.14921
L-Cysteine Hydrochloride	Traces	Traces
Leucine	6.677	6.1101
Lysine	0.9612	0.29927
Methionine	0.060984	0.23751
Phenylalanine	7.9042	5.94068
Proline	4.7299	0.9596
Serine	9.2875	0.19159
Threonine	11.3148	0.24195
Tryptophan	2.7106	2.24222
Tyrosine	1.303	1.84277
Valine	8.5757	6.50149
trans-Hydroxy L-Proline	Traces	Traces
Indole-3-Acetic Acid (IAA)	Traces	0.004247
trans-Zeatin	Traces	Traces
trans-Zeatin Riboside	Traces	Traces
Gibberellin	Traces	Traces
Total Lipids (g/100 mL)	0.0091	0.0072
Total Sugars (%)	0.042	0.039
Macro- and Micronutrients	mg L^−1^	
Nitrogen	330.8	386.8
Phosphorus	41.33	106.65
Potassium	750	27400
Calcium	19.8	28.4
Magnesium	25.6	52
Sulfur	46.3	86.9
Iron	51	77.3
Copper	0.03	0.04
Zinc	0.7	0.9
Manganese	106	133
Boron	0.6	1
Lead (g/L)	2.1	2.4

**Table 6:** Positive + negative ionization of *Egeria densa* extracts using ethanol and KCl with number of substances, average signal/noise (S/N), total chromatographic area (x106), average m/z (mass-to-charge ratio), and average retention time (RT).

Evaluations	Extraction	Average	STD ^1^
Number of substances	Ethanol	2358	24.417
Average S/N		476.475	20.305
Total chromatographic area (x106)		75.146	4.843
Average m/z ^2^		661.919	3.849
Average RT ^3^		39.038	0.351
Number of substances	KCL	3588	87.929
Average S/N		579.382	5.514
Total chromatographic area (x106)		78.491	1.958
Average m/z		802.694	1.758
Average RT		23.640	0.220

^1^—standard deviation; ^2^—mass-to-charge ratio; ^3^—retention time.

**Table 7:** Identification of species represented by treatment, species, and fresh matter doses used in the treatments.

Identification	Treatment	Dose (kg of Fresh Biomass/ha)
T1	Untreated control	-----
T2	*Egeria densa*	1 and 5
T3	*Ludwigia peploides*	1 and 5
T4	*Polygonum hydropiperoides*	1 and 5
T5	*Polygonum lapathifolium*	1 and 5
T6	*Eichhornia crassipes*	1 and 5
T7	*Hydrilla verticillata*	1 and 5
T8	*Alternanthera sessilis*	1 and 5
T9	*Commelina diffusa*	1 and 5
T10	*Brachiaria subquadripara*	1 and 5
T11	*Paspalum repens*	1 and 5
T12	*Enydra anagallis*	1 and 5
T13	*Typha domingensis*	1 and 5

**Table 8:** Identification of treatments, species, doses (kg of fresh matter), and extraction methods used in the applications.

Identification	Treatment	Dose (kg of Fresh Matter/ha)	Extraction
T1	Untreated control	-----	-----
T2	*Egeria densa*	0.25	92.8% Ethanol
T3	*Egeria densa*	0.5	92.8% Ethanol
T4	*Egeria densa*	1	92.8% Ethanol
T5	*Egeria densa*	2	92.8% Ethanol
T6	*Egeria densa*	4	92.8% Ethanol

## Data Availability

Data is unavailable due to privacy restrictions and patent protection.
